# FSCN1 Promotes Esophageal Carcinoma Progression Through Downregulating PTK6 *via* its RNA-Binding Protein Effect

**DOI:** 10.3389/fphar.2022.868296

**Published:** 2022-03-23

**Authors:** Hongfei Cai, Rui Wang, Ze Tang, Tianyu Lu, Youbin Cui

**Affiliations:** ^1^ Department of Thoracic Surgery, The First Hospital of Jilin University, Changchun, China; ^2^ Department of Epidemiology and Biostatistics, School of Public Health, Jilin University, Changchun, China

**Keywords:** Fscn1, PTK6, RBP, esophageal, carcinoma

## Abstract

**Objective:** Esophageal squamous cell carcinoma (ESCC) causes many deaths worldwide every year. Fascin actin-bundling protein 1(FSCN1) has been reported to be a promoter of ESCC via its actin-binding function, however, its new role as an RNA-binding protein (RBP) has not been investigated. Here, we explored the RBP role of FSCN1 in the development of ESCC.

**Methods:** Whole-genome expression sequencing was performed to screen for altered genes after FSCN1 knockdown. RNA immunoprecipitation was performed to determine the target mRNA of FSCN1 as an RBP. *In vitro* experiments with ECA-109 and KYSE-150 and *ex vivo* experiments in tumor-bearing mice were performed to investigate the effects of FSCN1 and Protein Tyrosine Kinase 6 (PTK6) on ESCC progression.

**Results:** FSCN1 could downregulate mRNA and the protein level of PTK6. The binding position of PTK6 (PTK6-T2) pre-mRNA to FSCN1 was determined. PTK6-T2 blocked the binding between FSCN1 and the pre-mRNA of PTK6, and thus reversed the promotion effect of FSCN1 on ESCC tumor progression via the AKT/GSK3β signaling pathway.

**Conclusion:** A novel effect of FSCN1, RBP-binding with the pre-mRNA of PTK6, was confirmed to play an important role in ESCC progression. PTK6-T2, which is a specific inhibitor of FSCN1 binding to the pre-mRNA of PTK6, could impede the development of ESCC.

## Introduction

Esophageal carcinoma (EC), which is the sixth leading cause of tumor-related death worldwide (Napier et al., 2014), comprises two main types: adenocarcinoma and esophageal squamous cell carcinoma (ESCC). ESCC is the predominant subtype (80–90%) of ECs in Asia and Western countries (Jemal et al., 2011; Arnold et al., 2015). Although multiple therapies have been developed in recent years owing to advanced-stage diagnosis, drug resistance, and highly occurring metastasis, the 5 year survival rate remains poor (15–25%) in ESCC patients (Chu et al., 2020; Jing et al., 2021). Hence, early-stage diagnosis and treatment of ESCC are crucial for prognosis.

The globular filamentous actin-binding protein FSCN1 (fascin actin-bundling protein 1), also known as fascin1 or fascin, stabilizes actin bundles, thereby mediating various cellular structures (Adams, 2004a; Hashimoto et al., 2011). FSCN1 is composed of four tandem fascin domains and supports lamellipodia, filopodia, microspikes, and other actin-based protrusions (Hashimoto et al., 2005). FSCN1 is related to numerous types of carcinomas, such as ESCC, bladder cancer, breast cancer, cervical cancer, and cholangiocarcinoma (Chiyomaru et al., 2010; Kano et al., 2010; Mao et al., 2016; Zhao et al., 2016; Wang et al., 2017; Ma and Li, 2019; Xu et al., 2021), which strongly suggests that FSCN1 might be a potential promising biomarker or therapeutic target.

In addition to studies on FSCN1 promoting the migration and invasion of tumor cells via its action-bundling activity (Liu et al., 2021), the formation of filopodia and invadopodia (Li et al., 2010; Machesky and Li, 2010), focal adhesion dynamics (Villari et al., 2015), and extracellular vesicle release (Clancy et al., 2019), numerous studies have also reported various upstream factors regulating FSCN1 in EC progression. For example, microRNA (miR)-145, miR-133a, miR-328, and miR326 target FSCN1 to suppress ESCC, and long non-coding RNA (lncRNA) LOC146880, lncRNA PVT1, lncRNA01711, and lncRNA00337 confer EC progression by inhibiting the miR/FSCN1 pathway (Kano et al., 2010; Akanuma et al., 2014; Shen et al., 2019; Shi et al., 2020; Tang et al., 2021; Xu et al., 2021). However, another interesting function of FSCN1, namely its action as an RNA-binding protein (RBP) (Castello et al., 2012), has not yet been studied. Here, we investigated the novel RNA-binding function of FSCN1 and found that FSCN1 inhibits protein tyrosine kinase 6 (PTK6) expression by binding to its pre-mRNA, thus facilitating tumor progression in ESCC.

## Materials and Methods

### Animal and Ethics Statement

All animal care and experimental protocols were performed in accordance with the recommendations of the Guide for the Care and Use of Laboratory Animals published by the US National Institutes of Health (NIH publication No. 8023, revised 1978), with the approval of the Ethics Committee of the First Hospital of Jilin University. BALB/c nu/nu nude mice were purchased from SIPEIFU Co., Ltd. (Beijing, China, 0205). All mice were housed in standard cages (4 mice per cage) in a specific pathogen-free environment and kept on a 12 h light/dark cycle with freely available food and water.

Approximately 1×10^6^ tumor cells were subcutaneously injected into each nude mouse. Tumor volume was measured on day 7 after tumor injection and every other day using a unified Vernier caliper. The tumor volume was calculated using a standard formula (length × width^2^ × 0.52). The mice were sacrificed 3 weeks after subcutaneous injection. The tumors were dissected for histological and immunohistochemical analyses.

### Cell Incubation

The human EC cell lines ECA-109 and KYSE-150 were purchased from the Cell Resource Center of the Chinese Academy of Sciences (Beijing, China) and cultured in RPMI-1640 medium (Gibco, TX, United States ) supplemented with 10% FBS (Gibco) and 1% penicillin–streptomycin. The cells were cultured, followed by trypsin digestion with trypsin/EDTA, and only cells grown to 80% confluence were seeded in 75 cm^2^ flasks. The cells were subcultured thrice weekly.

### RNA-Seq

RQ1 DNase (Promega, WI, United States ) was added to the total RNA to remove DNA. The quantity and quality of the extracted RNA were tested by measuring the absorbance at 260 nm/280 nm using SmartSpec Plus (Bio-Rad, CA, United States ). RNA integrity was verified by 1.5% agarose gel electrophoresis.

For RNA-seq library preparation, 1 μg of total RNA from each sample was used. For high-throughput sequencing, libraries were prepared following the manufacturer’s instructions and applied to the Illumina HiSeq X Ten system for 150 nt paired-end sequencing.

### FSCN1 and PTK6 Overexpression or Knockdown in Tumor Cells

The human EC cell lines ECA109 and KYSE-150 with stable FSCN1 and PTK6 overexpression (OE) or knockdown (KD) were established by transfection with lentivirus containing FSCN1-OE plasmid and PTK6-OE plasmid (pEZ-Lv105 vector; GenePharma, Shanghai, China) or FSCN1 short hairpin RNA (shRNA) and PTK6 shRNA (pGIPZ vector, GenePharma), respectively, followed by selection with puromycin (Invitrogen, Carlsbad, CA, United States ). Lipofectamine 3,000 reagent (Invitrogen, MA, United States ) was used for transient transfection of plasmids into ECA109 and KYSE-150 cells, according to the manufacturer’s instructions.

### RT-PCR Assay

Total RNA was extracted from tissues or cells using an RNeasy Mini Kit (74106; QIAGEN, Hilden, Germany) according to the manufacturer’s instructions. The extracted RNA was dissolved in RNase-free water, and the concentration of total RNA was determined using a spectrophotometer. RNA was then reverse-transcribed with a PrimeScript RT reagent kit with gDNA Eraser (TaKaRa, Shiga, Japan), and cDNA amplification was measured using SYBR Green PCR Master Mix (Roche, Basel, Switzerland) and a Light Cycler480 instrument (Roche). The data were normalized to the GAPDH expression levels in each individual sample. Quantitative values were obtained from the threshold cycle value (Ct), and relative mRNA expression levels were analyzed using the 2^−ΔΔCt^ method.

### Western Blotting Analysis

Proteins were extracted from cells or fresh tissue lysates from the treatment and experimental groups. Protein extracts were prepared in RIPA lysis buffer containing PMSF (100:1). A BCA protein assay kit (Cwbiotech, Beijing, China) was used to determine the protein concentration. Protein extracts were separated by 10% SDS-PAGE, transferred to PVDF membranes (Millipore, MA, United States ), blocked with 5% BSA for 1 h at room temperature, and then incubated with the corresponding primary antibodies overnight. Finally, the proteins were visualized using chemiluminescent horseradish peroxidase substrate (Millipore). The levels of target proteins, such as *β*-actin, were normalized to those of the loading control. Six independent experiments were performed to derive the mean values for statistical analysis. The primary antibodies used were rabbit anti-fascin antibody (1:1000, Abcam, Cambridge, UK, ab126772), rabbit anti-PTK6 antibody (1:1000, Abcam, ab233391), and rabbit anti-GAPDH antibody (1:1000, Cell Signaling Technology, MA, United States , 2118).

### Histology Examination and Immunohistochemical (IHC) Staining

At the end of the study, the tumor tissues were removed, fixed in 4% paraformaldehyde overnight, embedded in paraffin, and cut into 4 μm-thick cross sections. After deparaffinization, the paraffin sections were subjected to antigen retrieval with citrate buffer and treated with 3% H_2_O_2_ for 10 min at room temperature. The sections were then blocked with 5% non-immune goat serum for 30 min at 37°C and incubated with the corresponding primary antibodies at 4°C overnight. The primary antibodies used were rabbit anti-fascin antibody (1:1000, Abcam, ab126772) and rabbit anti-PTK6 antibody (1:1000, Abcam, ab233391). Horseradish peroxidase-conjugated secondary antibodies (PV-9001, ZSGB-Bio, Beijing, China) were added the next day for 30 min at 37°C, and a DAB kit (ZLI-9018, ZSGB-Bio) was used for color development according to the manufacturer’s instructions. Hematoxylin was used to counterstain the nuclei during IHC staining. Sections were incubated with non-immune IgG secondary antibody only, and no primary or secondary antibodies were used as negative controls.

### Cell Migration and Invasion Assay

Cancer cell migration was measured using 24-well Transwell plates (3,422, Corning, NY, United States ) with 8 μm-pore polycarbonate membranes. Tumor cells (5 × 10^5^ cells/mL) suspended in serum-free RPMI-1640 medium were grown in the upper chamber and incubated at 37°C for migration assays. Following 24 h of culture, the number of cells entering the lower chamber reflected the migration ability of tumor cells. Invasion assays were performed using membranes coated with Matrigel matrix (BD Biosciences, NJ, United States ). Cells must secrete matrix metalloproteinases in order to enter the lower chamber. Counting the number of cells entering the lower chamber could reflect the invasive ability of tumor cells. The cells were fixed with ice-cold methanol, stained with crystal violet (KGA229, KeyGEN Bio, Jiangsu, China), and then observed under an optical microscope (Nikon, Tokyo, Japan).

### Wound-Healing Assay

The migration ability of tumor cells was monitored using a wound-healing assay. Firstly, creating a “wound” in a cell monolayer, capturing the images during cell migration to close the wound, and comparing the images to quantify the migration rate of the cells. Treated cells were wounded using a stick. Detached cells were washed away and treated with the corresponding intervention. Images were captured 0 and 24 h after scratching. The healing area was analyzed using Image-Pro Plus 6.0.

### Cell Counting Kit-8 Assay

Cell viability was measured using the Cell Counting Kit-8 (CCK-8; C0038, Beyotime, Jiangsu, China), according to the manufacturer’s instructions. Absorbance at 450 nm was measured using a microplate reader (BioTek, VT, United States ).

### TUNEL Assay

Tumor cell apoptosis was detected by TUNEL using the *In Situ* Cell Death Detection Kit, TMR red (Roche), following the manufacturer’s instructions. The cells were cultured in 12-well plates and treated with the appropriate intervention. Following incubation for 24 h, cells were collected and fixed with 4% paraformaldehyde for 15 min at room temperature. The cells were then permeabilized with 0.1% Triton X-100 (T8200; Solarbio, Beijing, China) for 10 min. Cell nuclei were stained with DAPI (ab104139, Abcam) for 10 min at 37°C. The samples were observed under a fluorescence microscope with an excitation wavelength of 570–620 nm.

### EdU Assay

Cell proliferation was determined using the 5-ethynyl-2′-deoxyuridine (EdU) DNA Cell Proliferation Kit *in vitro* (C10310, RiboBio, Guangzhou, China) and *in vivo* (C0078S, Beyotime) according to the manufacturer’s instructions. *In vivo*, mice were injected i. p. with EdU (100 mg/kg) and sacrificed 4 h after injection. After the required treatment, the tumor cells were cultured with 50 μM EdU for 2 h. Then, the samples were fixed with 4% paraformaldehyde and incubated with Apollo dye solution to label proliferating cells. DAPI (ab104139, Abcam) was used to mark cell nuclei, and the samples were observed and photographed using a fluorescence microscope (Nikon).

### Statistical Analysis

All data are presented as mean ± SEM. Normality assumption of the data distribution was assessed using the Shapiro–Wilk test. For normally distributed data, statistical significance was assessed using Student’s *t*-test between two groups, or by one-way ANOVA between three or more groups. For data with a non-normal distribution, we performed a nonparametric statistical analysis using the Kruskal–Wallis test followed by the Dunn post hic test for multiple comparisons with one variable. In all statistical comparisons, *p* < 0.05 was considered statistically significant. SPSS version 20.0 (SPSS, IL, United States ) and GraphPad Prism version 8.0 software (GraphPad, CA, United States ) were used for statistical analyses.

## Results

### Whole-Genome Expression Sequencing After Downregulated FSCN1

**FIGURE 1:**
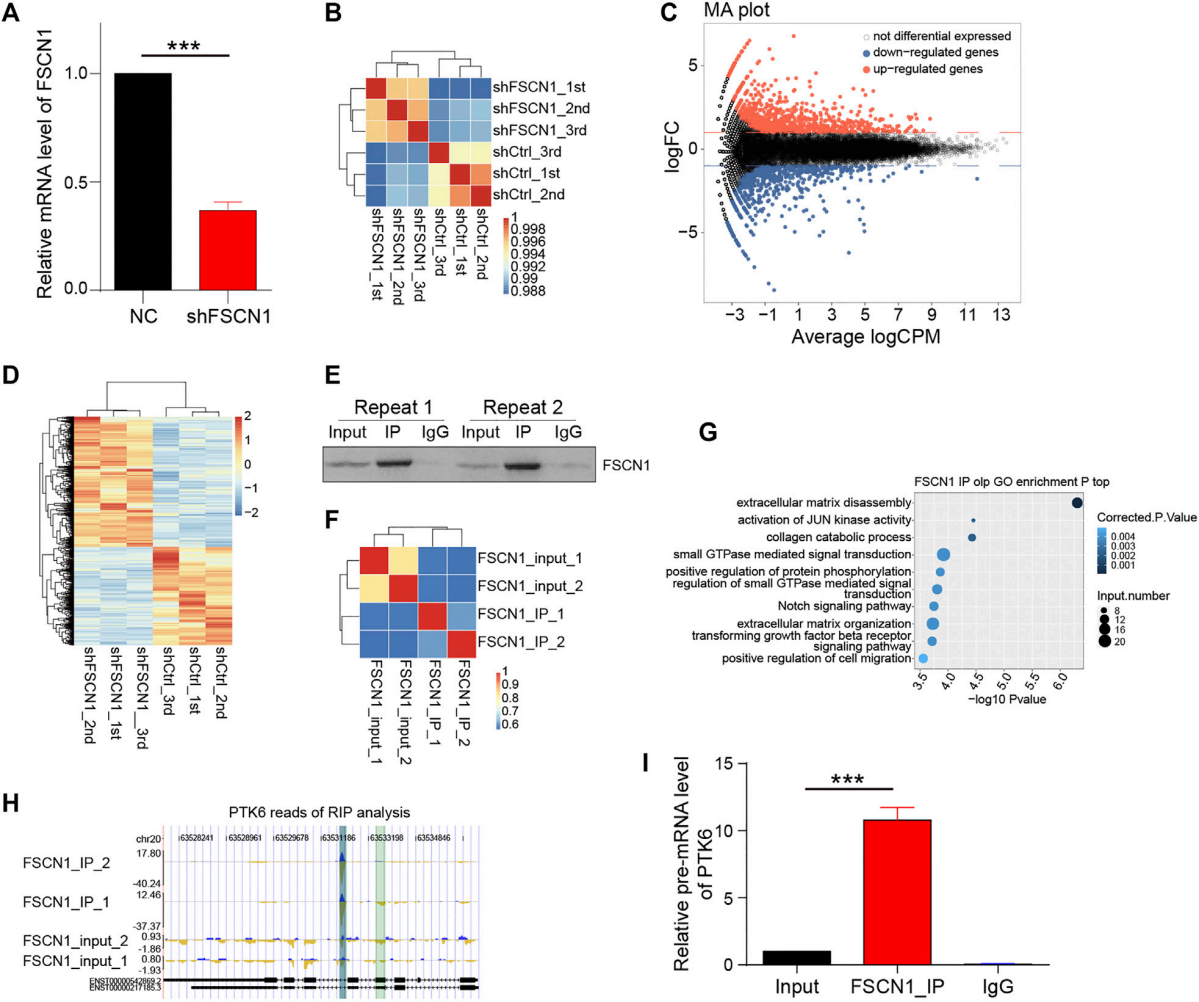
Whole-genome expression sequencing and RIP assay. **(A)** Two ECA-109 groups were transfected with negative control (NC) and shFSCN1 separately. Relative mRNA level of FSCN1 was detected. *n* = 3 per group, ***p < 0.001. **(B)** ECA-109 cells were transfected with NC (shCtrl) and shFSCN1 separately. The sample correlation among the control groups (shCtrl) and FSCN1 knock down groups (shFSCN1) is shown. **(C, D)** The differentially expressed genes (DEG) were analyzed and shown. **(E)** RNA immunoprecipitation (RIP) was performed by using FSCN1 monoclonal-antibody in ECA-109 cell lysate. The immunoprecipitation was detected by western blotting. **(F)** The sample correlation is shown. **(G)** Gene Ontology enrichment is shown in the sequencing analysis of RIP in **(F)**. **(H)** The peak reads of the sequence of PTK6 pulled down by FSCN1 in RIP analysis. **(I)** ECA-109 lysates were immunoprecipitated by FSCN1 antibody or IgG. Relative pre-mRNA level of PTK6 was detected. *n* = 3 per group, ***p < 0.001.

To explore the downstream molecules of FSCN1, shRNA targeting FSCN1 was designed and transfected into the ESCC cell line, ECA-109 (Figure 1A). Whole-genome expression profiling was performed to screen for related molecules. There was a good correlation between the samples in the three control (shCtrl) and three FSCN1 knockdown groups (shFSCN1, Figure 1B). Next, differentially expressed genes (DEGs) were analyzed. A total of 1036 upregulated and 781 downregulated genes were detected (Figure 1C). A heatmap of the DEGs is shown in Figure 1D. To investigate the RNA binding property of FSCN1 in ESCC, RNA immunoprecipitation (RIP) was performed using an FSCN1 monoclonal antibody in ECA-109 cell lysate, and the associated RNA was analyzed by RNA-seq (Figure 1E). Clustering analysis revealed considerable consistency between the two parallel repeats (Figure 1F). Based on the distribution and number of mRNA reads associated with FSCN1, 651 genes were screened. Moreover, Gene Ontology enrichment analysis showed that these genes primarily belonged to extracellular matrix disassembly, extracellular matrix organization, and cell migration (Figure 1G). The intersection among these 651 genes, 1817 DEGs after FSCN1 knockdown, and 1706 DEGs of EC from The Cancer Genome Atlas database were obtained. Finally, four genes were identified. Among them, only PTK6 was downregulated in EC tissues compared to that in para-carcinoma tissues. Further RIP analysis showed that the sequence of PTK6 could be pulled down by the immunoprecipitation of FSCN1, the peak of the reads located on chromosome 20 (Chr20), approximately 63531800–63532300 (Figure 1H), which fell into one of the introns of PTK6 (Chr20: 63532525–63530928, reverse strand). We then performed RIP analysis using the FSCN1 antibody again to confirm this intron sequence in the pre-mRNA of PTK6, and found that FSCN1 could effectively pull it down (Figure 1I), which suggested that FSCN1 might bind the intron of the pre-mRNA of PTK6 and perform certain functions in EC.

### FSCN1 Regulates PTK6 Expression in ESCC Cell Lines

**FIGURE 2:**
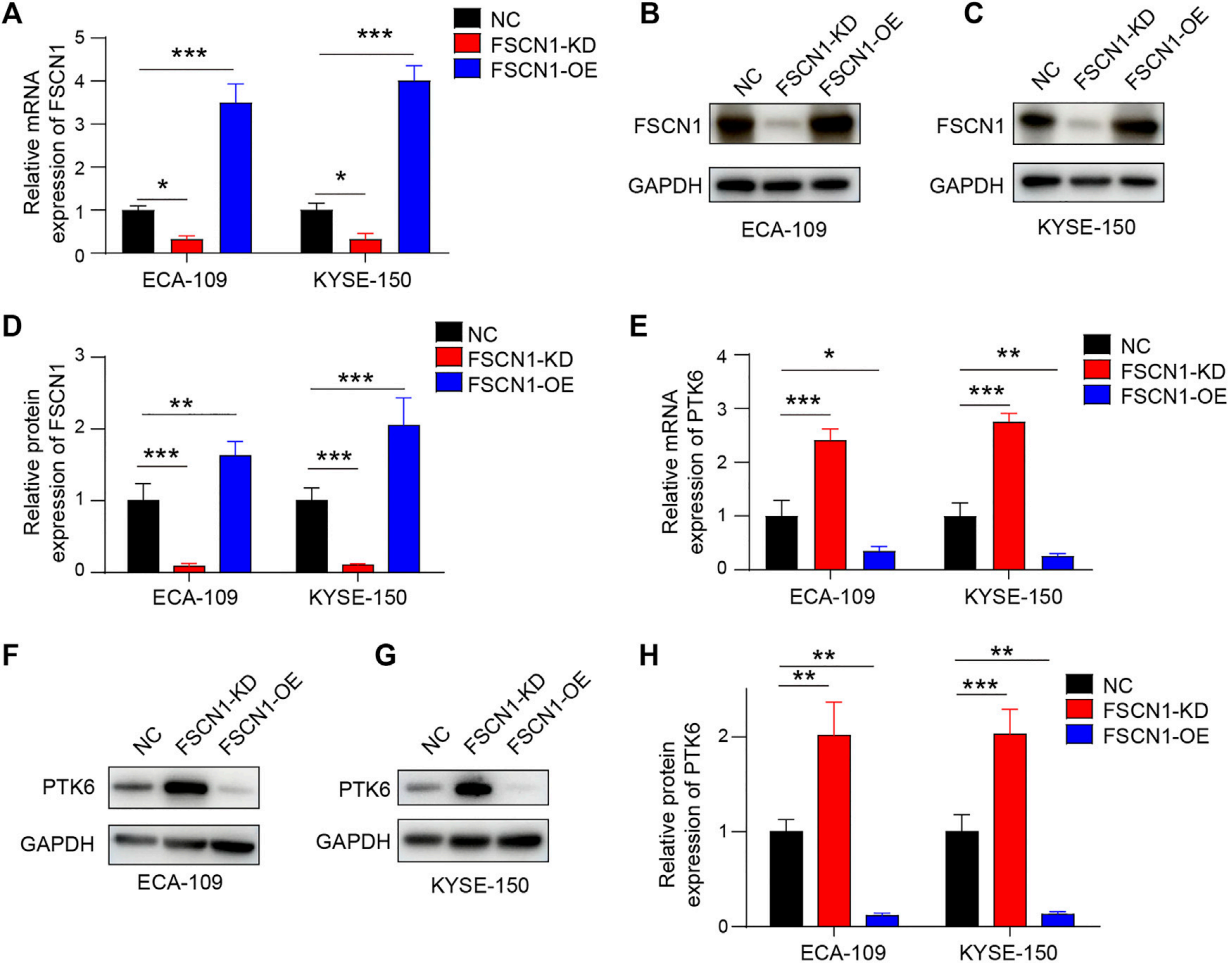
FSCN1 downregulates PTK6 expression in ESCC cell lines. **(A–D)** Lentivirus containing FSCN1 shRNA sequence (FSCN1-KD) and over-expressed sequence (FSCN1-OE) were added into ECA-109 and KYSE-150 mediums separately. Lentivirus expressing GFP only was used as a negative control (NC). The mRNA **(A)** and protein level **(B–D)** of FSCN1 in the 2 cell lines were detected. *n* = 3 per group, **p < 0.01, ***p < 0.001. **(E–H)** ECA-109 and KYSE-150 infected by NC, FSCN1-KD, and FSCN1-OE as indicated were harvested, and the mRNA **(E)** and protein level (F–H) of PTK6 in the 2 cell lines were detected. *n* = 3 per group, *p < 0.05, **p < 0.01, ***p < 0.001.

After screening for FSCN1 knockdown conditions in ECA-109 cells, we performed an experiment to confirm whether PTK6 was regulated by FSCN1. ECA-109 and KYSE-150 were chosen as ESCC cell lines. We infected these 2 cell lines with two lentiviruses containing the FSCN1 shRNA sequence (FSCN1-KD) and an overexpressed sequence (FSCN1-OE). The knockdown efficiency of FSCN1 mRNA was approximately 90%, and the overexpression efficiency was approximately 3–4-fold in ECA-109 and KYSE-150 cells (Figure 2A). Western blotting showed that the protein level of FSCN1 was knocked down in the FSCN1-KD group and overexpressed by approximately 4-fold in the FSCN1-OE group (Figures 2B–D). We then detected PTK6 mRNA and protein levels under different FSCN1 conditions and found that PTK6 was upregulated by FSCN1-KD and downregulated by FSCN1-OE at both mRNA and protein levels (Figures 2E–H). These results strongly suggest that both the mRNA and protein expression of PTK6 are regulated by FSCN1.

### The Binding Region of the Pre-mRNA of PTK6 With FSCN1 Located in the Intron

**FIGURE 3:**
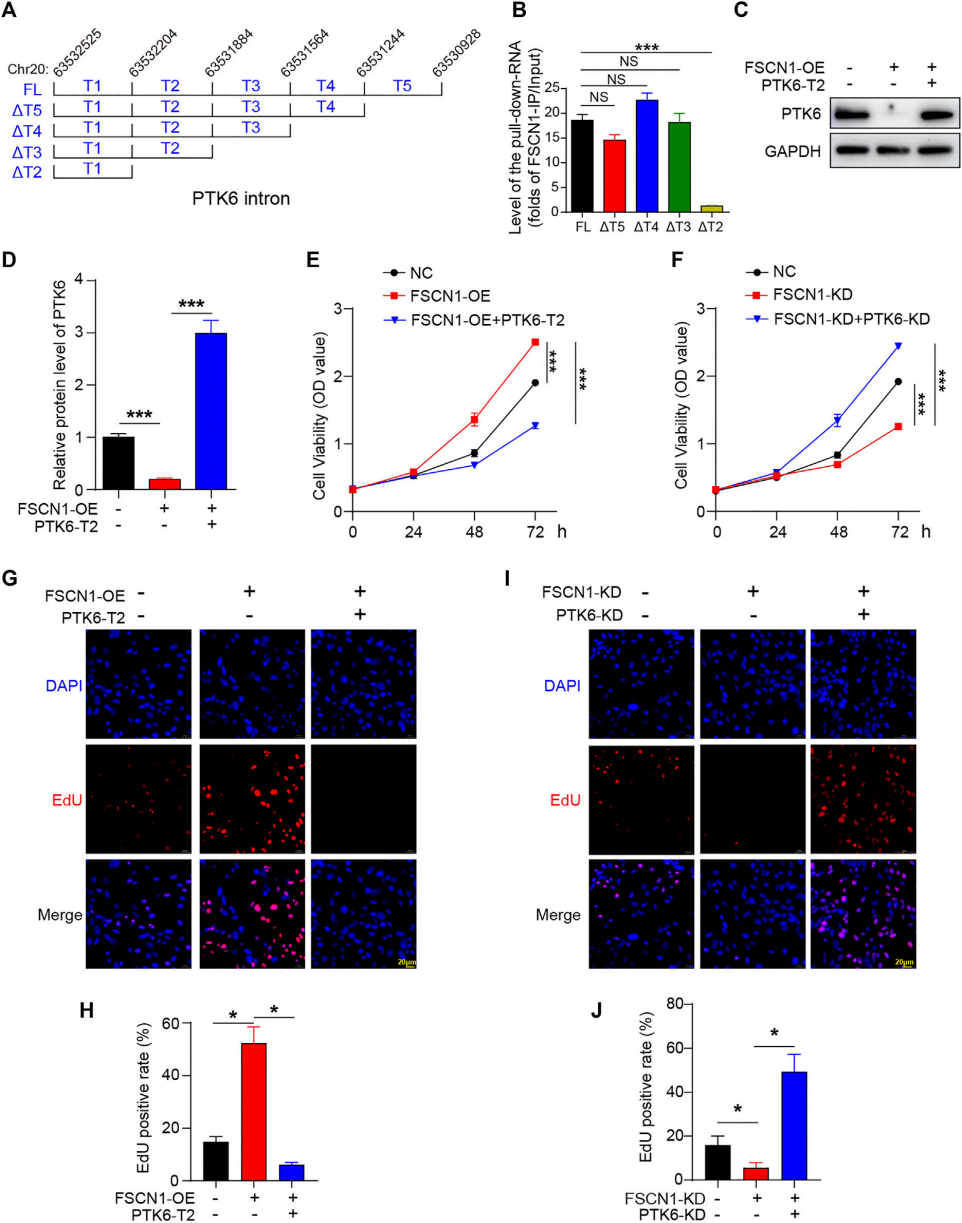
The binding region of the pre-mRNA of PTK6 with FSCN1 is PTK-T2 **(A)** Schematic diagram of the full length and truncated mutant of PTK6 intron located in chromosome 20. **(B)** Plasmids expressing PTK6-FL and five truncated mutants (ΔT5, ΔT4, ΔT3 and ΔT2) were transfected into ECA-109 for 48 h. RIP was performed using the FSCN1 antibody. The pull-down RNA level was detected. *n* = 3 per group, ***p < 0.001. **(C)** Cultured ECA-109 cells were infected with NC, FSCN1-OE, and FSCN1-OE + PTK6-T2 as indicated. The protein level of PTK6 was detected by western blotting **(D)** Statistical analysis of C. *n* = 3 per group, ***p < 0.001. **(E)** CCK-8 assay was performed in cultured ECA-109 cells infected with NC, FSCN1-OE, and FSCN1-OE + PTK6-T2 as indicated. *n* = 3 per group, ***p < 0.001. **(F)** CCK-8 assay was performed in cultured ECA-109 cells infected with NC, FSCN1-KD, and FSCN1-KD + PTK6-KD as indicated. *n* = 3 per group, ***p < 0.001. **(G)** ECA-109 cells expressing NC, FSCN1-OE, and FSCN1-OE + PTK6-T2 as indicated were stained by EdU. **(H)** Statistical analysis of G. *n* = 3 per group, *p < 0.05. **(I)** ECA-109 cells expressing NC, FSCN1-KD, and FSCN1-KD + PTK6-KD as indicated were stained by EdU. **(J)** Statistical analysis of I. *n* = 3 per group, *p < 0.05.

The FSCN1-binding intron of PTK6 is located on Chr20: 63532525–63530928, reverse strand. To confirm the exact binding of this intron to FSCN1, we constructed full-length and five truncated mutants (ΔT5, ΔT4, ΔT3, and ΔT2) and performed RIP analysis with FSCN1 IP in ECA-109 (Figure 3A). The results clearly showed that when the T2 region of this intron was lost, FSCN1 could not pull down RNA, suggesting that T2 is the region associated with FSCN1 (Figure 3B). We constructed a lentivirus expressing the T2 region of the pre-mRNA of PTK6 (PTK6-T2) as an antagonist to inhibit the binding with FSCN1 and found that this lentivirus could upregulate the protein level of PTK6 (Figures 3C, D), which suggested that FSCN1 inhibited PTK6 expression by binding with the pre-mRNA.

### FSCN1 Facilitates ECA-109 Proliferation via Inhibiting PTK6

To investigate whether FSCN1 affects tumor cell viability and proliferation via PTK6, a lentivirus expressing shRNA targeting PTK6 (PTK6-KD) was constructed. The lentivirus expressing GFP was used as a negative control (NC). We then infected ECA-109 cells with the indicated lentiviruses to explore whether FSCN1 affected cell proliferation via PTK6. The CCK-8 assay showed that FSCN1-OE facilitated the viability of ECA-109 cells, however, PTK6-T2 clearly inhibited this effect (Figure 3E). The opposite effect was observed in FSCN1-KD and PTK6-KD cells (Figure 3F). An EdU staining assay was performed to detect the proliferation state. The results showed that FSCN1-OE promoted proliferation, whereas PTK6-T2 changed this effect (Figures 3G, H). FSCN1-KD decreased EdU staining, while PTK6-KD increased it, even under FSCN1 knockdown conditions (Figures 3I, J). These results suggest that FSCN1 facilitates ECA-109 cell proliferation via regulating PTK6.

### FSCN1 Inhibits ECA-109 Cell Apoptosis *via* Inhibiting PTK6

**FIGURE 4:**
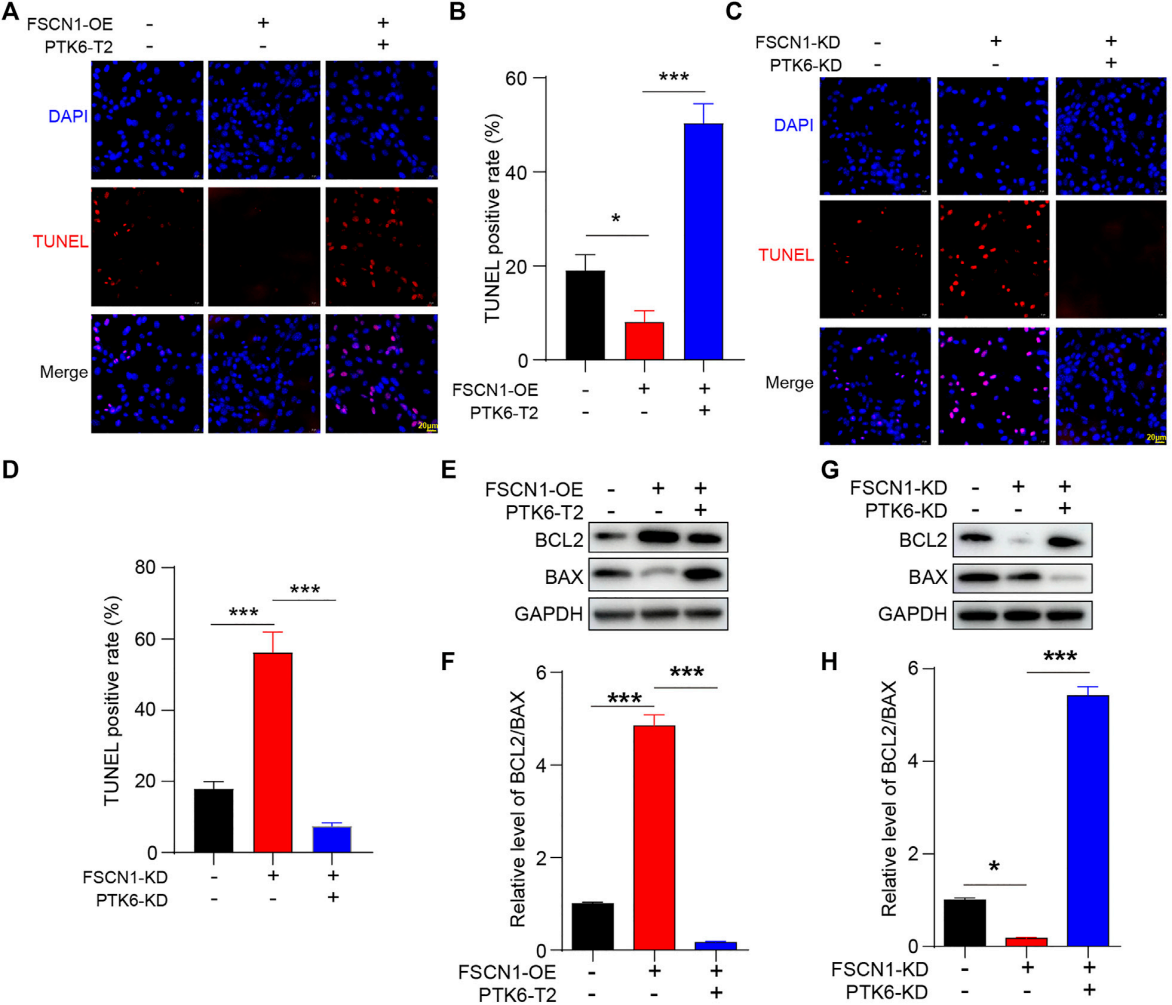
PTK-T2 increases ECA-109 apoptosis. **(A)** ECA-109 cells expressing NC, FSCN1-OE, and FSCN1-OE + PTK6-T2 as indicated were stained by TUNEL. **(B)** Statistical analysis of A. *n* = 3 per group, *p < 0.05, ***p < 0.001. **(C)** ECA-109 cells expressing NC, FSCN1-KD, and FSCN1-KD + PTK6-KD as indicated were stained by TUNEL. **(D)** Statistical analysis of C. *n* = 3 per group, ***p < 0.001. **(E)** ECA-109 cells expressing NC, FSCN1-OE, and FSCN1-OE + PTK6-T2 as indicated were harvested. BCL2 and BAX were detected by western blotting. **(F)** Statistical analysis of E. n = 3 per group, ***p < 0.001. **(G)** ECA-109 cells expressing NC, FSCN1-KD, and FSCN1-KD + PTK6-KD as indicated were harvested. BCL2 and BAX were detected by western blotting. **(H)** Statistical analysis of G. *n* = 3 per group, *p < 0.05, ***p < 0.001.

Lentiviruses of FSCN1-OE, FSCN1-KD, PTK6-T2, and PTK6-KD were also added to infect ECA-109 cells to study their influence on apoptosis. TUNEL staining showed that FSCN1-OE significantly alleviated the TUNEL signal, whereas PTK6-T2 promoted it under starvation conditions (Figures 4A, B). FSCN1 knockdown increased apoptosis (Figures 4C, D). The classical apoptosis pathway proteins BCL2 and BAX were also detected, showing that FSCN1 increased the anti-apoptotic BCL2, while PTK6 could inhibit it (Figures 4E–H). These results strongly suggest that FSCN1 inhibits apoptosis, PTK6 promotes apoptosis, and acts downstream of FSCN1.

### FSCN1 Promotes the Migration, Invasion, and Colony Formation of ECA-109 *via* Inhibiting PTK6

**FIGURE 5:**
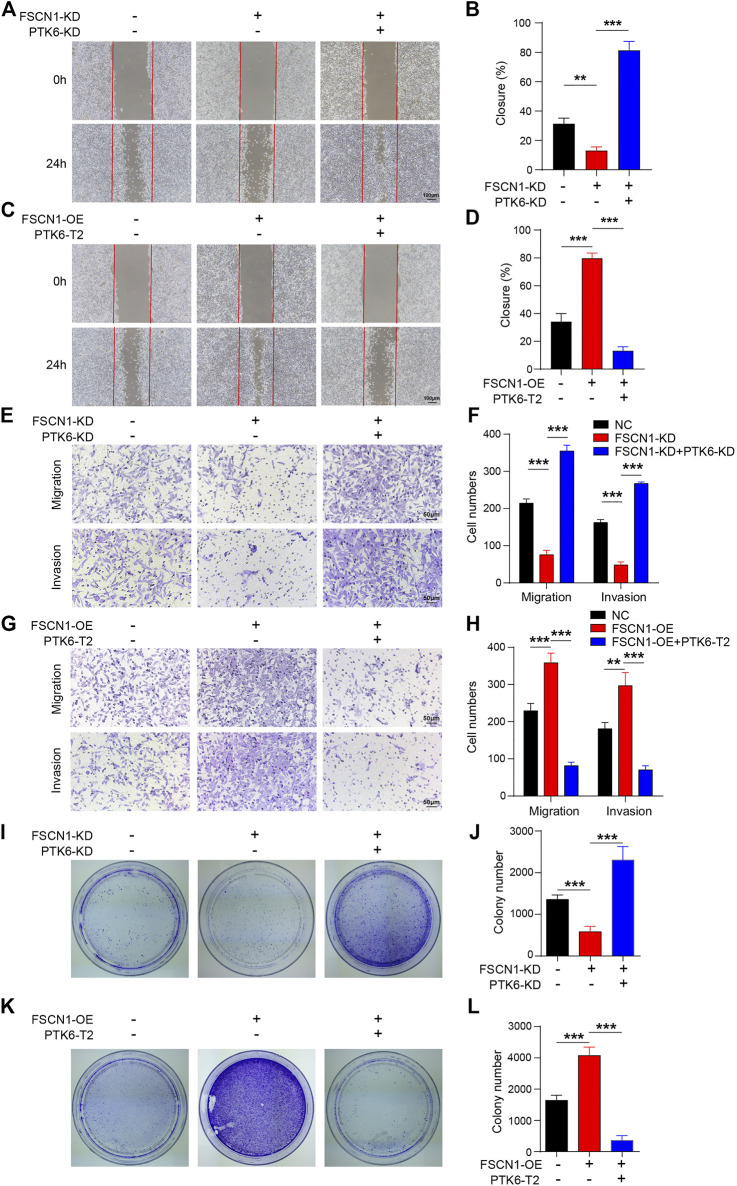
PTK-T2 inhibits ECA-109 migration and invasion. **(A)** The scratch test was performed using ECA-109 cells expressing NC, FSCN1-KD, and FSCN1-KD + PTK6-KD as indicated. **(B)** Statistical analysis of A. *n* = 3 per group, **p < 0.01, ***p < 0.001. **(C)** The scratch test was performed using ECA-109 cells expressing NC, FSCN1-OE, and FSCN1-OE + PTK6-T2 as indicated. **(D)** Statistical analysis of C. *n* = 3 per group, ***p < 0.001. **(E)** Transwell and Matrigel tests were performed using ECA-109 cells expressing NC, FSCN1-KD, and FSCN1-KD + PTK6-KD as indicated. **(F)** Statistical analysis of E. *n* = 3 per group, ***p < 0.001. **(G)** The Transwell and Matrigel tests were performed using ECA-109 cells expressing NC, FSCN1-OE, and FSCN1-OE + PTK6-T2 as indicated. **(H)** Statistical analysis of G. n = 3 per group, **p < 0.01, ***p < 0.001. **(I)** Colony formation of ECA-109 cells expressing NC, FSCN1-KD, and FSCN1-KD + PTK6-KD, as indicated, were performed. **(J)** Statistical analysis of I. *n* = 3 per group, ***p < 0.001. **(K)** Colony formation of ECA-109 cells expressing NC, FSCN1-OE, and FSCN1-OE + PTK6-T2 as indicated were performed. **(L)** Statistical analysis of K. *n* = 3 per group, ***p < 0.001.

The migration, invasion, and colony formation of ECA-109 cells were detected under FSCN1 and PTK6 knockdown and overexpression conditions. ECA-109 cells from different groups were infected with lentiviruses expressing FSCN1-OE, FSCN1-KD, PTK6-T2, or PTK6-KD, as indicated. The scratch test revealed that FSCN1 could promote migration; however, binding with the mRNA of PTK6 played an essential role (Figures 5A–D). Moreover, the Transwell and Matrigel tests showed that the enhancement of FSCN1 on the migration and invasion of ECA-109 cells predominantly relied on the inhibition of PTK6 (Figures 5E–H). The plate clonality assay revealed that FSCN1 increased colony formation, and PTK6 inhibited colony formation (Figures5I–L). These results suggest that FSCN1 promotes the migration, invasion, and colony formation of ECA-109 cells by inhibiting PTK6.

### AKT and GSK3β Are the Downstream Pathways of FSCN1-PTK6

**FIGURE 6:**
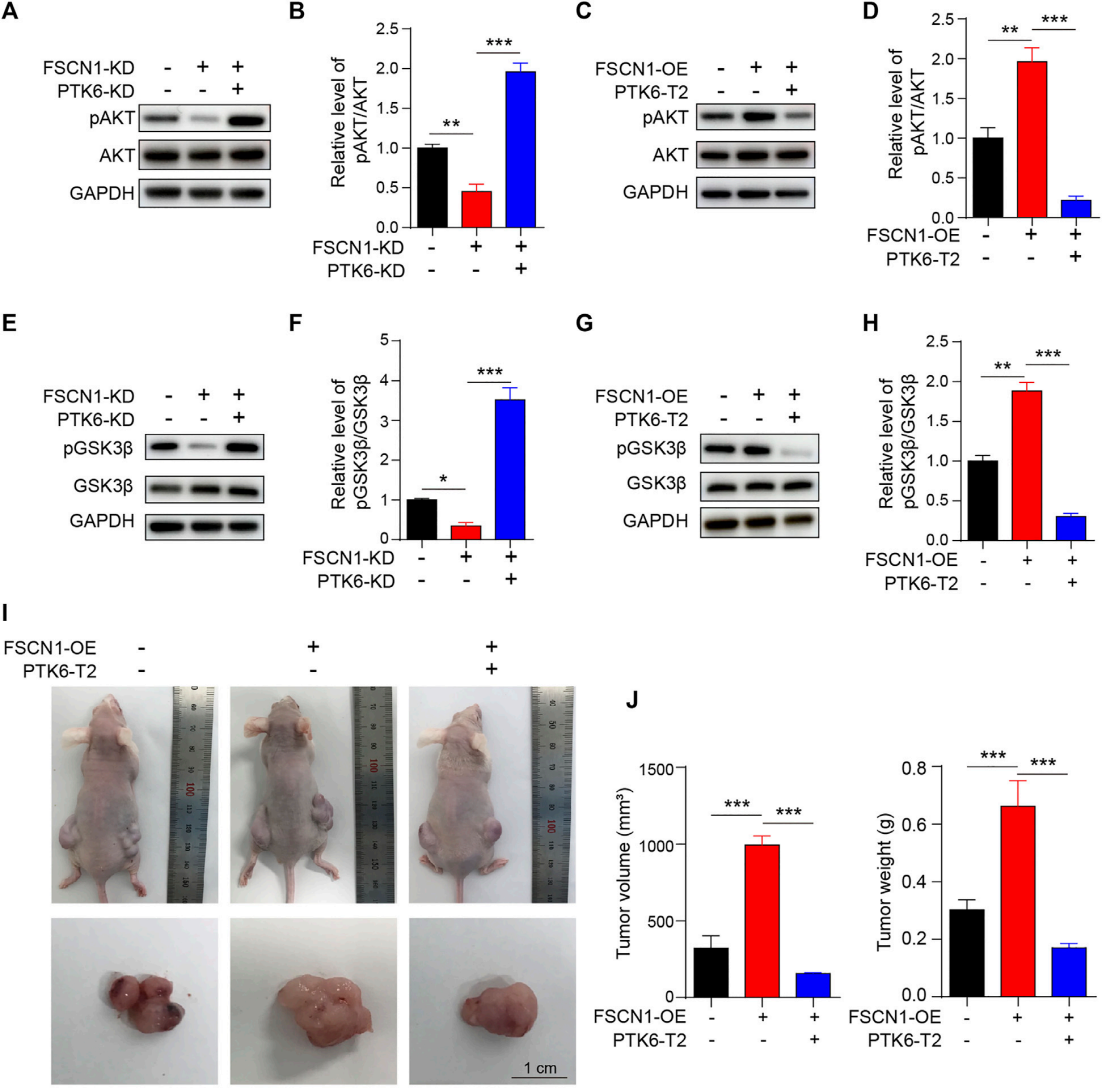
AKT and GSK3β are the downstream pathways of FSCN1-PTK6 signaling. **(A)** ECA-109 cells expressing NC, FSCN1-KD, and FSCN1-KD + PTK6-KD as indicated were harvested. AKT and pAKT were detected by western blotting. **(B)** Statistical analysis of A. *n* = 3 per group, **p < 0.01, ***p < 0.001. **(C)** ECA-109 cells expressing NC, FSCN1-OE, and FSCN1-OE + PTK6-T2, as indicated, were harvested. AKT and pAKT were detected by western blotting. **(D)** Statistical analysis of C. *n* = 3 per group, **p < 0.01, ***p < 0.001. **(E)** ECA-109 cells expressing NC, FSCN1-KD, and FSCN1-KD + PTK6-KD, as indicated, were harvested. GSK3β and p GSK3β were detected by western blotting. **(F)** Statistical analysis of E. *n* = 3 per group, *p < 0.05, ***p < 0.001. **(G)** ECA-109 cells expressing NC, FSCN1-OE, and FSCN1-OE + PTK6-T2, as indicated, were harvested. GSK3β and pGSK3β were detected by western blotting. **(H)** Statistical analysis of G. *n* = 3 per group, **p < 0.01, ***p < 0.001. **(I)** Three stable clones of ECA-109 cell lines of NC, only FSCN1-OE expressing, and both PTK6-T2 and FSCN1-OE expressing were generated via lentiviral system. Then the three types of ECA-109 were injected into nude mice to generate the ECA-109 tumor-bearing model. Mice and tumors were shown. **(J)** Statistical analysis of the tumor volume and weight of I. *n* = 3 per group, ***p < 0.001.

AKT and GSK3β are downstream signaling pathways of PTK6 (Ma et al., 2012). Hence, we detected the phosphorylation levels of these two proteins (pAKT and pGSK3β) under FSCN1-OE, FSCN1-KD, PTK6-T2, or PTK6-KD conditions, as indicated. As shown in Figures 6A–D, pAKT and pGSK3β are downregulated by FSCN1-KD, however, they can be rescued by PTK6-KD. FSCN1-OE promoted pAKT and pGSK3β levels, while PTK6-T2 suppressed them (Figures 6E–H). These results strongly suggest that the binding of FSCN1 to PTK6 mRNA could enhance the phosphorylation of AKT and GSK3β, thereby facilitating the progression of ESCC.

### FSCN1 Promotes *ex vivo* Tumor Growth *via* Binding With the mRNA of PTK6 in Tumor-Bearing Mice

**FIGURE 7:**
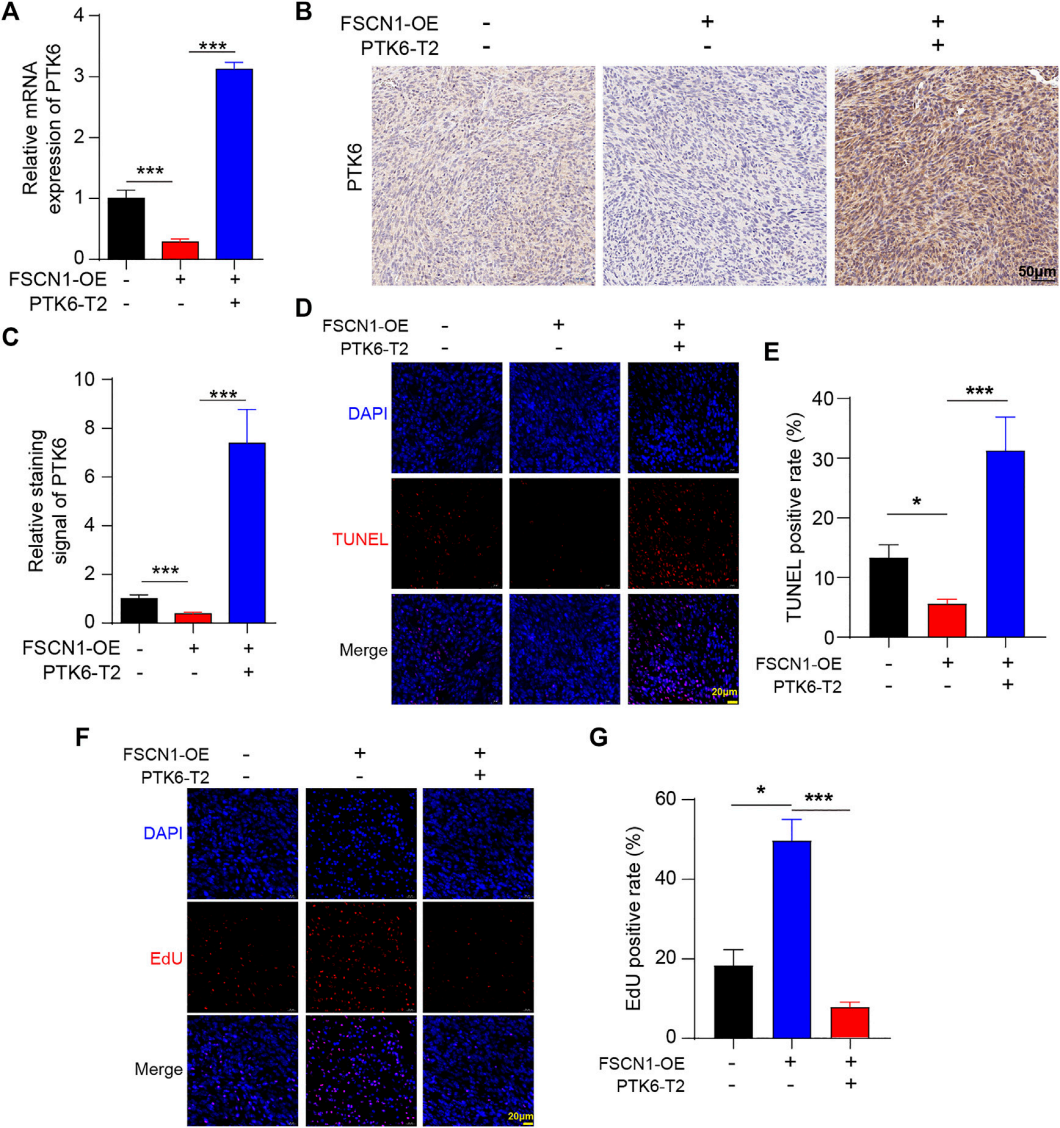
PTK-T2 inhibits ESCC tumor progression *ex vivo*. **(A)** The mRNA level of PTK6 in tumor tissues of NC, FSCN1-OE, and FSCN1-OE + PTK6-T2 groups was detected. *n* = 5 per group, ***p < 0.001. **(B)** PTK6 in tumor tissues of NC, FSCN1-OE, and FSCN1-OE + PTK6-T2 groups was detected by immunohistochemical staining (**C**) Statistical analysis of B. *n* = 5 per group, ***p < 0.001. **(D)** TUNEL assay was performed in tumor tissues of NC, FSCN1-OE, and FSCN1-OE + PTK6-T2 groups. **(E)** Statistical analysis of D. *n* = 5 per group, *p < 0.05, ***p < 0.001. **(F)** EdU assay was performed in tumor tissues of NC, FSCN1-OE, and FSCN1-OE + PTK6-T2 groups. **(G)** Statistical analysis of F. *n* = 5 per group, *p < 0.05, ***p < 0.001.

**FIGURE 8:**
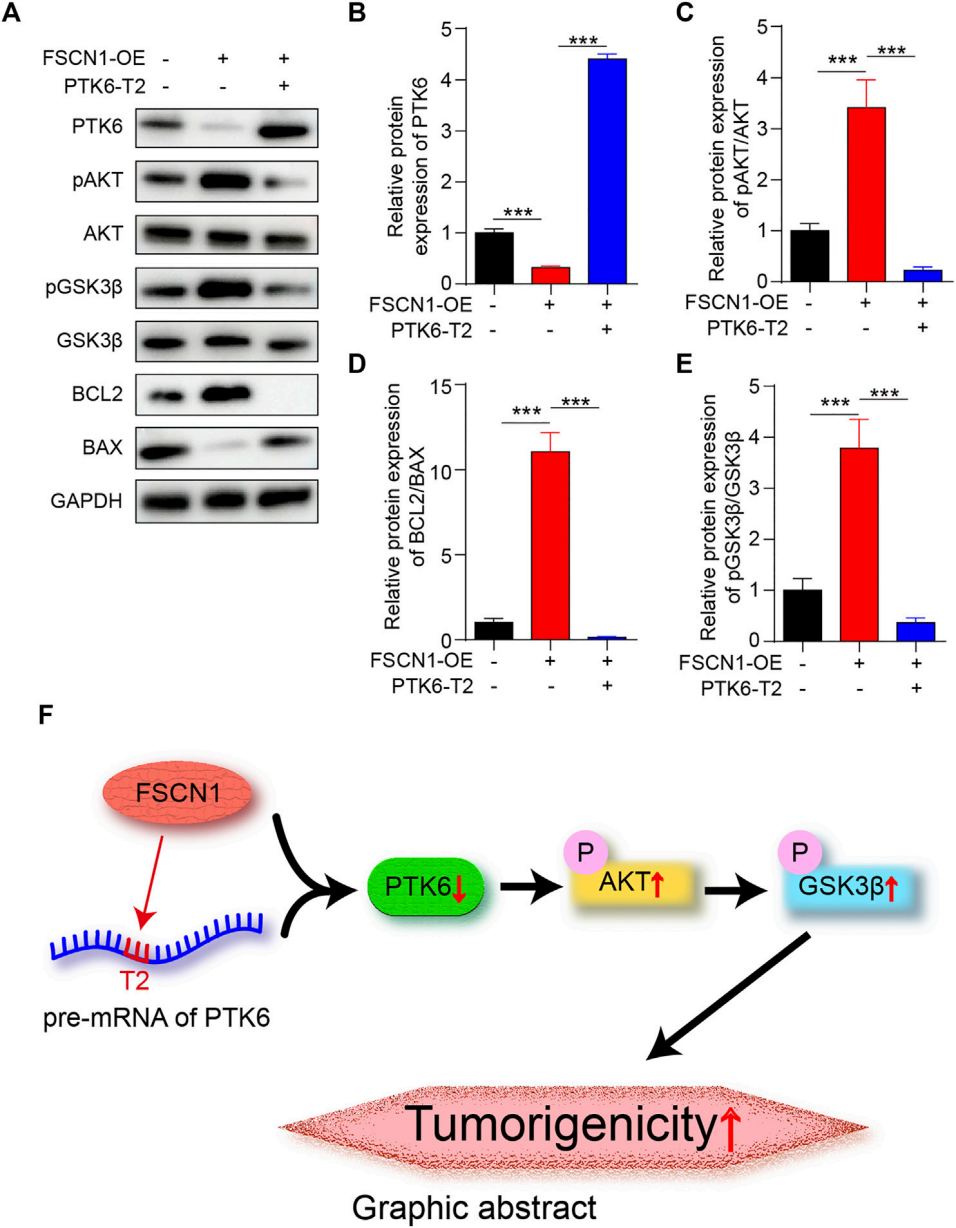
AKT and GSK3β are the downstream pathways of PTK6 *ex vivo*. **(A)** The protein levels of PTK6, AKT, pAKT, GSK3β, pGSK3β, BCL2, and BAX in tumor tissues of NC, FSCN1-OE, and FSCN1-OE + PTK6-T2 groups was detected by western blotting. **(B–E)** Statistical analysis of A. *n* = 5 per group, ***p < 0.001. **(F)** Graphic abstract: FSCN1 performs the RBP effect, binds with pre-mRNA of PTK6, inhibits PTK6 protein level, and thus upregulates the downstream AKT/GSK3β signaling pathway, which plays an important role in EC progression.

To further confirm the role of FSCN1 and PTK6 in ESCC progression, we established three types of ECA-109 cell lines of NC, only FSCN1-OE expressing, and both PTK6-T2 and FSCN1-OE expressing separately. Then, the three types of ECA-109 were injected into nude mice to generate the ECA-109 tumor-bearing model (Figure 6I). The tumor volume and weight of the FSCN1-OE group were significantly larger than those of the control group, whereas they were substantially smaller in the PTK6-T2 group (Figure 6J), suggesting that FSCN1 promotes EC growth by inhibiting PTK6. PTK6 mRNA in tumor tissue was detected and exhibited the same tendency as in the *in vitro* experiments (Figure 7A). IHC staining of PTK6 showed that PTK6 was clearly inhibited in the FSCN1-OE group but increased when PTK6-T2 was present (Figures 7B, C). TUNEL and EdU were also detected in the tumor tissues, which showed that apoptosis was inhibited by FSCN1-OE but promoted by PTK6-T2, and proliferation was facilitated by FSNC1-OE but restrained by PTK6-T2 (Figures 7D–G). pAKT, pGSK3β, BCL2, and BAX were also detected and showed the same tendency as *in vitro* cultured ECA-109 cells (Figures 8A–E). These results suggest that FSCN1 promotes tumor growth by binding to PTK6 mRNA and activating the AKT and GSK3β pathways.

## Discussion

The key finding of this study was that FSCN1 plays an RBP role in targeting PTK6 by promoting the AKT signaling pathway to facilitate the EC progression. FSCN1 is an evolutionarily conserved protein whose primary function is to bind actin into tight, relatively rigid, parallel bundles (Edwards and Bryan, 1995). The actin-bundling activity of FSCN1 is responsible for multiple cellular physiological processes, for example, cell adhesion, motility, migration, and cellular interactions (Adams, 2004a; Adams, 2004b; Jayo and Parsons, 2010). In addition, other cellular physiological processes, such as extracellular vesicle release (Beghein et al., 2018), focal adhesion dynamics (Villari et al., 2015), cancer cell stemness (Barnawi et al., 2016; Kang et al., 2018; Barnawi et al., 2020), histone methylation, and gene transcription (Saad et al., 2016) are independent of the actin-bundling activity of FSCN1.

PTK6, also known as breast tumor kinase, was originally identified in human breast tumors (Mitchell et al., 1994). PTK6 is low to undetectable in normal tissues and is highly expressed in numerous tumors, such as breast, cervix, and ovary (Schmandt et al., 2006; Ito et al., 2016; Wang et al., 2016; Ang et al., 2021). In ESCC, PTK6 expression was found to be lower in tumors than in peritumoral esophageal tissues (Chen et al., 2014). However, there are no reports on the relationship between FSCN1 and PTK6.

The prognostic value of FSCN1 expression has been demonstrated in several cancer types, and its upregulation is considered to promote migration and invasion (Liu et al., 2021). The potential RBP function of FSCN1 has been previously reported, however no further studies have been conducted. In the present study, we constructed an shRNA targeting FSCN1 in ECA-109 cells and screened PTK6, a novel target of FSCN1. Further investigation showed that the mRNA of PTK6 could be pulled down by FSCN1. This is the first evidence of FSCN1 acting as an RBP. We then confirmed that FSCN1 inhibited PTK6 expression in ECA-109 and KYSE-150 cells. Further investigation of the binding site of FSCN1 and PTK6 mRNA showed that FSCN1 binds to the intron of the pre-mRNA of PTK6. We screened the location on the intron (PTK6-T2) and expressed it as an inhibitor of FSCN1-PTK6 binding. We found that PTK6-T2 blocked the binding of FSCN1 to PTK6 pre-mRNA. Thus, it reversed the tumor promoting effect of FSCN1 in ESCC. Finally, we expressed PTK6-T2 in tumor-bearing mice and confirmed this effect in *ex vivo* experiments.

In conclusion, we confirmed a novel effect of FSCN1: RBP-binding with pre-mRNA of PTK6 and the downstream AKT/GSK3β signaling pathway, which plays an important role in EC progression. PTK6-T2, which acts as a specific inhibitor of FSCN1 binding to the pre-mRNA of PTK6, might be developed into a new therapy for tumor treatment (Figure 8F).

## Data Availability

The datasets presented in this study can be found in online repositories. The names of the repository/repositories and accession number(s) can be found below: National Center for Biotechnology Information (NCBI) Bio Project database under accession numbers: GSE197621 and GSE197624.
